# Progress in Polyhedral Oligomeric Silsesquioxane (POSS) Photoresists: A Comprehensive Review across Lithographic Systems

**DOI:** 10.3390/polym16060846

**Published:** 2024-03-19

**Authors:** Zaoxia Wen, Xingyu Liu, Wenxiu Chen, Ruolin Zhou, Hao Wu, Yongmei Xia, Lianbin Wu

**Affiliations:** College of Material Chemistry and Chemical Engineering, Key Laboratory of Organosilicon Chemistry and Material Technology of Zhejiang Province, Key Laboratory of Organosilicon Material Technology, Ministry of Education, Hangzhou Normal University, Hangzhou 311121, China; 2021111017017@stu.hznu.edu.cn (Z.W.); 2021111017019@stu.hznu.edu.cn (X.L.); 2022111009087@stu.hznu.edu.cn (W.C.); 2022112009082@stu.hznu.edu.cn (R.Z.); 2022112009066@stu.hznu.edu.cn (H.W.); 2022112009100@stu.hznu.edu.cn (Y.X.)

**Keywords:** photoresist resin, polyhedral oligomeric silsesquioxane (POSS), POSS-based photoresist, lithographic systems

## Abstract

This paper offers a comprehensive overview of the polyhedral oligomeric silsesquioxane (POSS) and POSS-based composites within the realm of photoresist resin. The study involves a systematic exploration and discussion of the contributions made by POSS across various lithographic systems, with specific emphasis on critical parameters such as film formation, sensitivity, resolution, solubility, and edge roughness. These lithographic systems encompass X-ray lithography (XRL), deep ultraviolet nanoimprint lithography (DUV-NIL), extreme ultraviolet lithography (EUV), and guided self-assembled lithography (DSA). The principal objective of this paper is to furnish valuable insights into the development and utilization of POSS-based photoresist materials in diverse lithographic contexts.

## 1. Introduction

**Figure 1:**
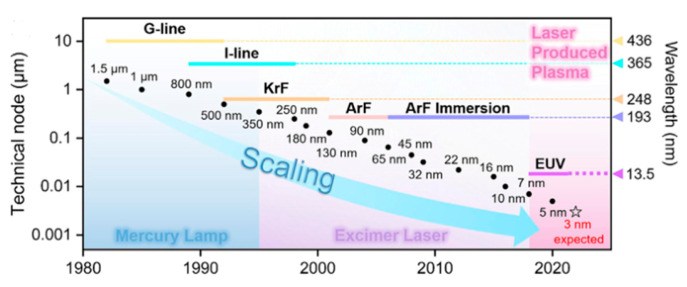
Year of Introduction. Adapted from Ref. [7] with permission from Elsevier Ltd.

With the rapid advancement of integrated circuits, there is a continual reduction in the characteristic size of semiconductor chips. As depicted in Figure 1, the characteristic size has been reduced from 436 nm (G-line) to 365 nm (UV), to 248 nm (deep ultraviolet (DUV) KrF, 193 nm (ArF excimer lasers), and to even smaller sizes of 13.5 nm and 3–5 nm (extreme ultraviolet (EUV) [1]. The photoresist, a pivotal material affecting the characteristic size of chips, is subject to increasingly stringent demands regarding its sensitivity, resolution, and line edge roughness, among other properties [2,3]. Typically, a photoresist comprises a film-forming polymer, a photosensitive component, and a solvent. Its overall performance is contingent upon characteristics such as dissolution behavior, film formation, etch resistance, thermodynamics, and more [4,5,6].

For instance, current usage of cyclized rubber photoresists results in the formation of cross-linked network structures during the lithographic process, endowing them with resistance to developing solutions, excellent adhesion, and etch resistance. However, a reduction in pattern resolution occurs because developers penetrate the exposure area [8]. In EUV lithography, organic photoresists exhibit limitations in resolution, sensitivity, and line edge roughness due to low radiation yield, inadequate corrosion resistance, and high absorption rates [9,10]. To address these issues, researchers have explored inorganic materials as photoresist components [11,12]. Hydrogen silsesquioxane (HSQ) is an excellent inorganic material, with heat resistance, chemical corrosion resistance, and high transparency of siloxane, and the potential for chemical modification to regulate its stability in solvents. The cage structure of HSQ opened under the action of the electron beam, leading to hydrogen emission and the eventual formation of a cross-linked network structure [13,14]. The HSQ exhibited a resolution lower than 10 nm and a lower dielectric constant, facilitating its application in optoelectronic products [15,16,17].

**Figure 2:**
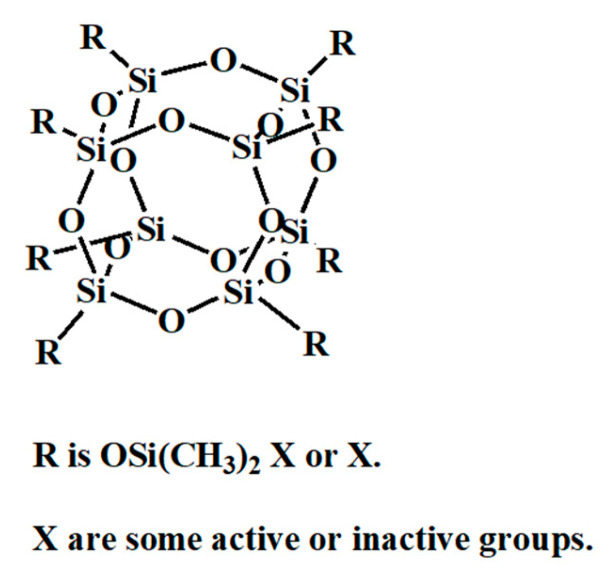
The chemical structure of the POSS.

Polyhedral oligomeric silsesquioxane (POSS) is an organic-inorganic hybrid compound characterized by a substantial silicon component coupled with chemically modified external organic moieties, including hydrogen, alkyl, alkene, and aryl groups. These organic groups may not be identical. When the organic groups are all H, it is referred to as HSQ. Notably, POSS exhibits a diminutive nanoscale rigid structure, typically ranging from 1 to 3 nm, centered around a Si-O-Si core (see Figure 2), which contrasts with the larger size of conventional polymer photoresists typically spanning from 3 to 5 nm [18,19,20,21,22].

The versatility of POSS is manifested through its capacity for construction via both chemical and physical methods, resulting in an extensive array of species [23]. These species find applications across diverse domains, including modifications of phenolic resin, epoxy resin, and phosphate resin [24,25,26,27]. The incorporation of POSS into composite materials imparts enhanced properties, such as improved dielectric characteristics, flame retardancy, and thermodynamic attributes [28,29,30]. Another notable attribute of POSS is its amenability to chemical modification, thereby enhancing its solubility in organic solvents and facilitating dispersion within substrates [31,32,33,34,35].

Furthermore, POSS can function as either a main chain or a side group, forming covalent bonds within synthetic composite materials. Such hybrid materials exhibit heightened stability and bolstered overall performance [36,37]. For instance, H. M. Lin and colleagues developed a photocured copolymer comprising POSS and methacrylate components as a negative photoresist resin [38]. This formulation achieved a remarkable sensitivity of 10.8 mJ·cm^−2^ and exhibited a glass transition temperature (*T*_g_) of 139 °C, surpassing the sensitivity and *T*_g_ values of pure methacrylate-based counterparts (71.8 mJ·cm^−2^ and 85.3 °C, respectively). Consequently, POSS has emerged as a focal point of interest in the realm of photoresist development. In light of this, the present study undertakes a comprehensive literature review, examining the performance of POSS in lithography employing diverse light sources. The objective is to contribute positively to the advancement of photoresist technology and the wider application of POSS in this domain.

## 2. Classification of the POSS-Base Photoresist

### 2.1. The EBL-POSS-Based Photoresist

Electron Beam Lithography (EBL) is a precision technique that employs electron beams to delineate nanoscale patterns on the surface of a photoresist resin-coated film with exceptional accuracy. One of its distinguishing features lies in the capability to modulate the electron beam dosage, making it highly versatile. The resultant exposure effects bear resemblance to those achieved through Extreme Ultraviolet (EUV) lithography, potentially offering an alternative to EUV light sources in lithographic applications [39].

Traditional photoresist polymers, characterized by lower glass transition temperatures (*T*_g_) and larger molecular dimensions, are plagued by pattern collapse and inadequate line edge roughness, which pose impediments to achieving finer photoresist patterns [40]. For instance, in the case of PMMA, line width fluctuations exceeding 3 nm are observed even when resolution requirements are met, resulting in the degradation of lithographic patterns [41]. In response to the challenges posed by chain entanglement and aggregation of PMMA during lithography, H. Namatsu et al. explored the pattern properties of Hydrogen Silsesquioxane (HSQ) at 70 kV electron beam exposure and using 2.38% tetramethyl ammonium hydroxide (TMAH) as the developer in 1998 [42]. HSQ’s unique three-dimensional framework impedes easy diffusion and entanglement (involving Si-H bond breaking and polymerization) upon electron beam exposure, resulting in notable sensitivity of 300 μC·cm^−2^ and contrast values of 5, respectively, when normalized to a film thickness of 0.8 (*D*_0.8_). Furthermore, line width fluctuations were confined to less than 2 nm. The exceptional performance of HSQ in EBL may be attributed to its dissolution rate in alkaline solutions, cross-linking stability induced by electron beam exposure, and the role of the developer TMAH [43,44].

**Figure 3:**
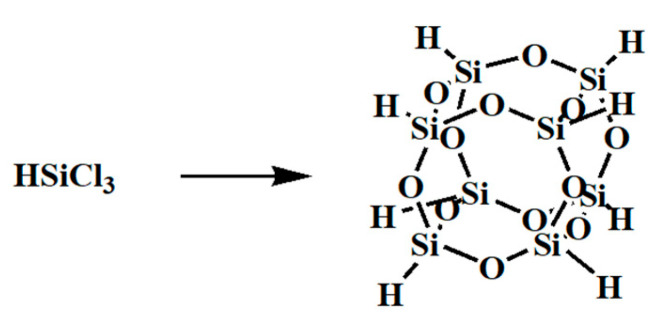
HSQ by hydrocondensation of chlorosilane.

As an exemplar of EBL-POSS-based photoresist, HSQ has garnered significant attention from researchers. HSQ is characterized by its straightforward synthesis as an inorganic material (see Figure 3), comprising solely Si-O and Si-H bonds, with the Si-O bond exhibiting superior strength over Si-H bonds, making the latter more prone to activation. In 2003, Michael J. and colleagues harnessed EBL technology to expose HSQ films, achieving line widths as narrow as 6 nm (using 50 kV e-beam on HSQ films of thickness 30 nm) and periodic gratings spaced at 27 nm [45]. A comprehensive review conducted by A. E. Grigorescu in 2009 underscored the influence of exposure, baking, development, and storage on HSQ in EBL lithography, highlighting that HSQ lithography remained an evolving field [46]. HSQ, serving as a negative photoresist material, mitigates secondary scattering during EBL, thus reducing the impact of the electron beam on the inhibitor film. It boasts high *T*_g_ and nanoscale molecular dimensions [47]. However, HSQ grapples with issues concerning low sensitivity and long-term chemical stability [48].

**Figure 4:**
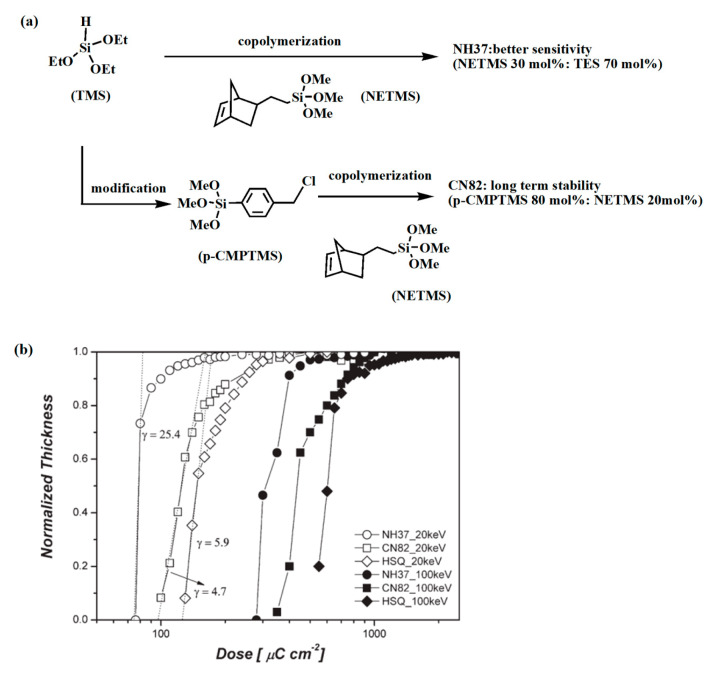
(**a**) Synthesis scheme of high sensitivity and high stability; (**b**) Contrast curves of HSQ and the novel silicone polymers under 20 and 100 keV electron beam irradiation, respectively, subsequently developed in TMAH 25 wt%, solution. Adapted from Ref. [49] with permission from the American Chemical Society.

**Figure 5:**
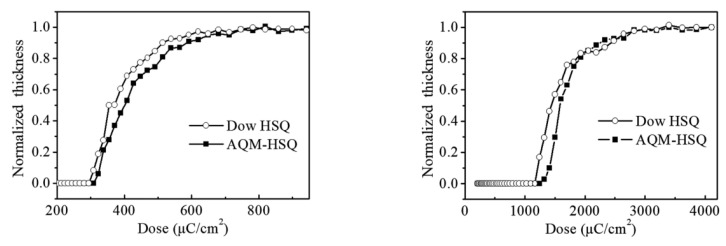
Contrast curves after AQM-HSQ and Dow HSQ using 25% TMAH (**left**); AQM-HSQ and Dow HSQ (**right**). Adapted from Ref. [50] with permission from The American Vacuum Society.

In 2010, Jae Hwan Sim and colleagues sought to enhance HSQ sensitivity and stability while preserving resolution and line edge roughness by introducing diaphragm and chloromethyl phenyl groups (see Figure 4) [49]. In 2019, Shen et al. [50] explored the performance distinctions between applied quantum materials-HSQ (AQM-HSQ) and Dow HSQ in two different developers (TMAH and salt developer). Their findings revealed that Dow HSQ exhibited greater sensitivity in TMAH developers, whereas the contrast displayed the opposite trend (see Figure 5). Furthermore, the electron beam dose in EBL plays a pivotal role in meeting distinct pattern requirements, as evidenced by HSQ’s capability to etch 20 nm patterns after exposure to 4000 μC·cm^−2^. Word et al. conducted experiments involving spin-coated HSQ films with thicknesses of 40 nm and 30 nm. They varied the EBL lithography dose and observed line widths ranging from 7 to 20 nm [45].

**Figure 6:**
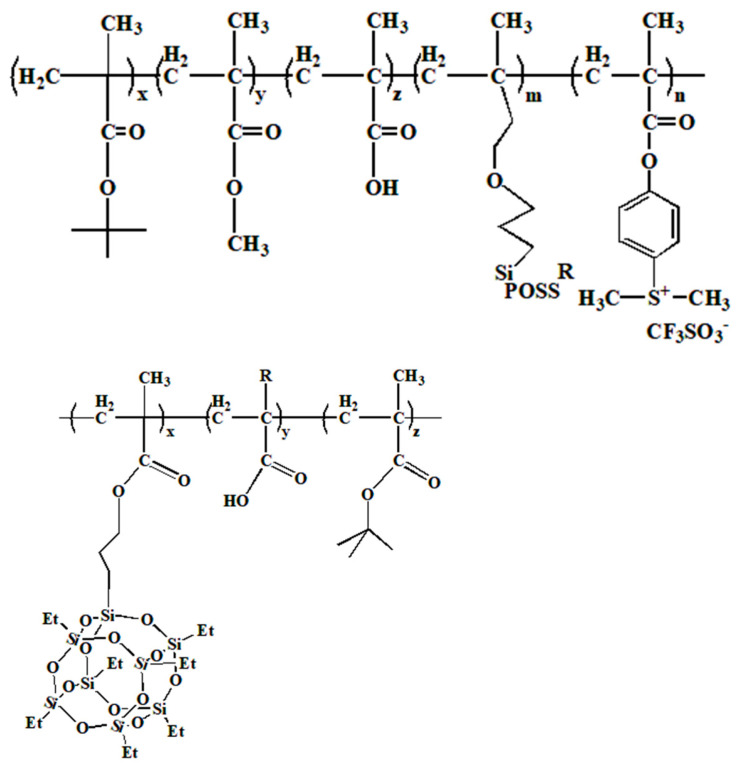
Microstructure of the nanocomposite resist (NanoRT-3b): Some contain fluorine POSS-based (methyl) acrylate ternary copolymer.

The prominent EBL-POSS-based photoresist, typified by HSQ, exhibits noteworthy etching resistance and affords flexibility in adjusting resolution and sensitivity as required. Furthermore, by incorporating POSS as a side group into other resin matrices and introducing photosensitive acid-producing agents (PAG) into the main chain of these resins, it becomes possible to bolster the etching resistance of the resin, expedite EBL polymerization and extend the range of electron beam dose suitable for EBL lithography [51]. In particular, the grafting POSS into fluorinated acrylic resin effectively addressed the problem of weak adhesion and apparent phase separation between methacrylic resins and silicon wafers (see Figure 6) [52,53].

While HSQ has established itself as a prominent contender in EBL technology, it falls short of enabling lithographic patterns with higher sensitivity and resolution compared to the current semiconductor market demands. Notably, achieving patterns below 5 nm presents challenges, as higher resolution patterns become challenging due to the proximity effect inherent to EBL [43,54]. Addressing such challenges, scholars have explored the use of Kr^26+^ as an exposure source, yielding a smaller feature size for HSQ. This development provides valuable insights for the future advancement of EBL-POSS-based photoresists [55].

The good EBL properties of HSQ are extremely important for the development of POSS as a photoresist resin. On the one hand, EBL not only has extremely high resolution and accuracy but also has high versatility compared to other technologies like EUV lithography, NIL, and X-ray lithography [56,57]. On the other hand, HSQ has the smallest nanomolecular size in a series of POSS, along with high thermal stability, high etching resistance, low dielectric constant, and good film formation. Consequently, HSQ is able to form lithography patterns with high-resolution and low-edge roughness. However, there are some challenging problems in the practical application of HSQ. Firstly, the chemical stability [48]. In the storage process, HSQ is prone to contamination by water, oxygen, and other substances. The contamination leads to great changes in molecular size through its secondary polymerization, resulting in the decrease of lithography sensitivity and increased pattern line edge thickness. Moreover, the lithographic mechanism of HSQ is still unclear. Although the mechanism of HSQ by EBL lithography has been proposed, the mechanism of EUV lithography is still unclear. This hinders the assessment of POSS as a photoresist [13,58]. Finally, there are sustainability issues of EBL-POSS-based photoresists, such as HSQ undergoing hydrolysis and condensation with chlorosilane, and the adhesion level of silicon-based photoresists and the silicon wafer is not uniform. For low adhesion, hexamethyldisilazane (HMDS) is used to treat the silicon wafer surface. Like traditional photoresist resin, EBL-POSS-based photoresist still requires a strong alkaline developer in the development process, which poses risks to human health and environmental sustainability. Therefore, further exploration is needed to enhance HSQ as a photoresist resin.

### 2.2. The X-ray POSS-Based Photoresist

X-ray lithography is a technology employed for patterning masks using short-wave X-rays. It finds its primary application in fabricating structures with high aspect ratios, particularly for small device lithography. Notably, X-ray lithography is characterized by minimal diffraction effects. Originally proposed by the Massachusetts Institute of Technology [59], X-ray lithography complements EBL in the manufacturing of submicron devices [60]. A comprehensive review of the development of X-ray lithography by Juan R. Maldonado and colleagues in 2016 exists in the literature [61]. Therefore, it will not be reiterated here.

In the fabrication of high-resolution X-ray lithography and even deep X-ray lithography, the quality requirements for photoresists are stringent, primarily aimed at preventing structural defects post-exposure [62,63]. In 1990, Hiroshi Ban et al. synthesized acetylated phenylsilsesquioxane oligomer (APSQ) through acetylation reactions using phenylsesquioxane as a raw material. The polymer comprising APSQ and photosensitizer initially achieved a line width of 1.7 μm in X-ray lithography [64]. In the realm of X-ray photoresists, the derivative known as X-ray-POSS-based photoresist has emerged as a promising option.

**Figure 7:**
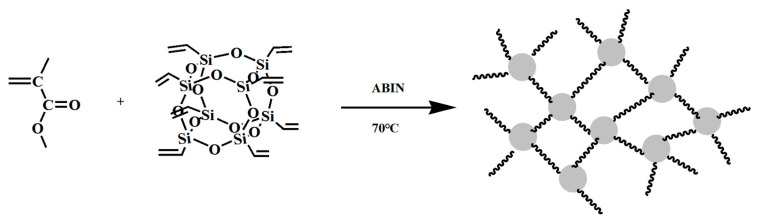
Schematic diagram of the mesh polymer formation of methyl methacrylate with octavinyl polysemisiloxane.

**Figure 8:**
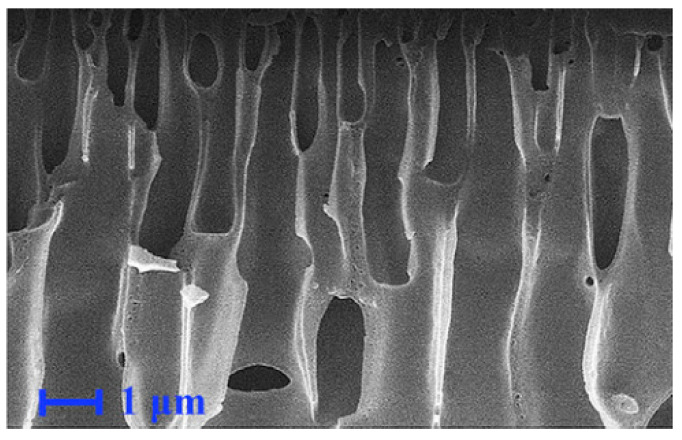
SEM image of PMMA-POSS films prepared by reverse rotation. Adapted from Ref. [65] with permission from Elsevier B.V.

The PMMA-POSS-based photoresist has exhibited significant promise in X-ray lithography, capitalizing on the combined strengths of PMMA and POSS. The PMMA-POSS formulation incorporates H-POSS or vinyl in place of octamer half-siloxane (Vi-POSS) and methacrylic acid (MA) as raw materials. This leads to silicon hydrogen addition reactions or radical polymerization, culminating in the formation of a cross-linked network structure. As illustrated in Figure 7, an example of mesh polymer formation utilizing methyl methacrylate (MMA) and Vi-POSS is presented [65]. This approach enhances the weather resistance, thermal stability, solubility, and *T*_g_ of the polymer. However, it also introduces new challenges, such as the presence of a porous structure within the polymer, resulting in a significant amount of free volume and inhibiting the degree of crosslinking in the product [66]. Nonetheless, the PMMA-POSS copolymer, produced through a counter-rotating method, does not exhibit surface cracking, addressing the fragility of the PMMA film. This improvement is attributed to the introduction of -COOH and -OH groups, which enhance the adhesion and hydrophobicity of the polymer material on the silicon substrate. However, scanning electron microscopy (SEM) reveals pore defects in the polymer film (as shown in Figure 8), which adversely affect its performance as a photoresist resin [65].

The utilization of PMMA-POSS as a photoresist resin has encountered certain challenges. To address these issues, researchers have undertaken innovative approaches, including the development of distinct polymer configurations such as mesh-type, graft-chain-type, and remote-claw-type structures [67]. Gonsalves K. E. and colleagues conducted research involving the incorporation of MMA into a toluene solution of POSS, followed by the addition of tert-butyl acrylate (TBA). This approach resulted in the synthesis of various ratios of POSS/MMA polymers (as detailed in Table 1) and subsequent exploration of the properties exhibited by the POSS/MMA series polymers when subjected to X-ray lithography [47]. The authors observed that these polymers demonstrated favorable characteristics, dissolving readily in azodiisonitrile. They exhibited excellent film-forming capabilities, strong adhesion to silicon wafers, and highly effective development in X-ray lithography. Remarkably, the film thickness remained relatively unaffected, and these polymers displayed a remarkable contrast value of 23.5 and an impressive sensitivity of 1350 mJ·cm^−2^.

During the 1980s and 1990s, X-ray lithography garnered significant attention as a promising lithographic technique, particularly in the context of next-generation lithography [61,68]. However, the utilization of photoresists that incorporate POSS in conjunction with X-ray exposure has received relatively limited research attention. This is attributed to the intricate interplay of various factors, including thermal effects and signal integrity, which influence the feasibility and efficacy of such combinations. Exploring the potential of POSS-derived compounds as X-ray photoresist materials represents a crucial avenue of support for X-ray chip technology. This research endeavor holds the promise of significantly enhancing the economic viability of lithographic technology, ultimately contributing to advancements in the field.

### 2.3. The UV-NIL POSS-Based Photoresist

**Figure 9:**
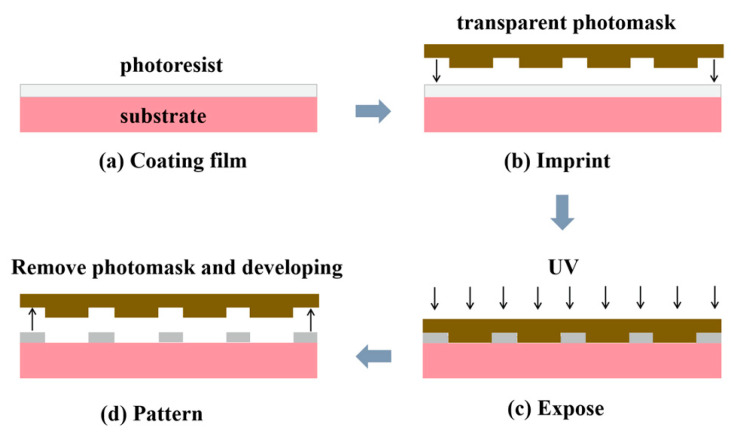
Flow diagram of UV-NIL lithography.

The fundamental principle of UV-NIL lies in the utilization of ultraviolet (UV) light to nanopattern, a photoresist resin film that has been applied onto a substrate, resulting in the creation of a finely patterned mold. In this process, following the deposition of the photoresist onto the substrate, the nanopatterned mold is pressed into the photoresist material. Subsequently, the photoresist fills the mold and is cured through UV light exposure, ultimately yielding the desired nanopattern. A schematic representation of the UV-NIL process is illustrated in Figure 9 [69], where the impression pattern is replicated within the resist and subsequently transferred onto the substrate through plasma etching. UV-NIL boasts distinctive attributes, including ultra-high resolution, elevated yield rates, minimal pattern damage, and cost-effectiveness [70]. Achieving lithographic patterns with sub-10 nm resolutions necessitates precise control over the close contact between the photoresist and the mold [71,72].

**Figure 10:**
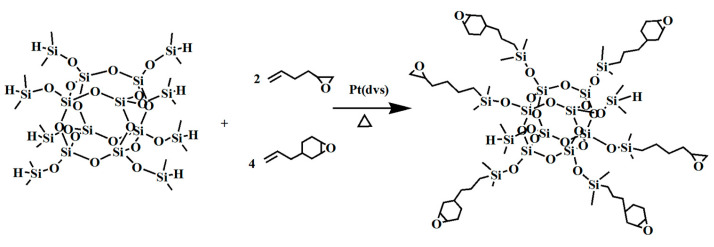
Epoxy-functionalized-POSS.

Presently, methods employed for etching nanoparticles into intricate patterns predominantly encompass techniques such as high-temperature film sintering and the use of commercial photoresist dispersions [73,74]. However, these etching procedures are often associated with drawbacks such as substantial free radical generation, pronounced volume shrinkage, and coarse pattern surfaces [75,76]. POSS derivatives have emerged as valuable tools for enhancing film stability, tailoring surface hydrophobicity, and mitigating pattern defects. Notably, in 2011, Nikolaos Kehagias et al. [77] employed a silicon hydrogen addition reaction to synthesize epoxy-functionalized POSS (as depicted in Figure 10) and subsequently investigated the NIL performance of this hybrid material on silicon substrates. The reactive epoxy group cross-linked after UV exposure, producing polymers with higher thermal and mechanical properties and a lower volume shrinkage, demonstrating the good application of this material in NIL. In 2017, Shu Jiang et al. [78] achieved the fabrication of nanopillar patterns with resolutions ranging from 50 to 100 nm through NIL, utilizing HSQ films and PDMS molds, all without the application of external pressure. In 2005, Tao et al. [79] reported the successful application of PMMA-POSS in duplex NIL processes. Moreover, HSQ has found an application in UV-NIL procedures [80]. Furthermore, sulfhydryl functionalization-POSS (SH-POSS), diazenone-POSS, and PMMA-POSS each exhibit distinct performance advantages within the realm of UV-NIL.

**Figure 11:**
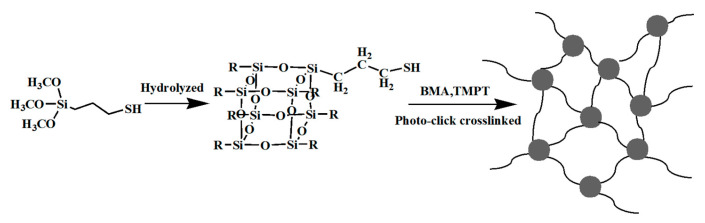

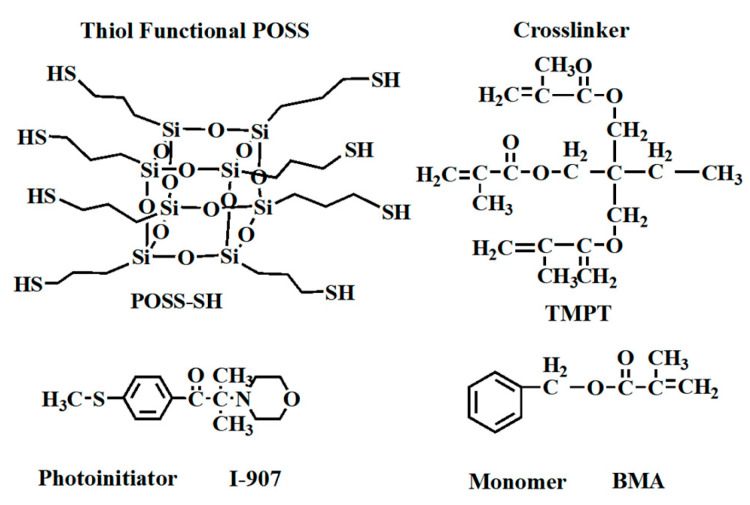
Schematic diagram of POSS-SH preparation by trimethylsilane and doping of BMA and TMPT.

SH-POSS exhibits notable properties, including corrosion resistance, high-temperature tolerance, and minimal volume shrinkage rate [81]. Figure 11 illustrates the formation of a cross-linked compound through the integration of SH-POSS and trihydroxymethylpropane triacrylate (TMPT), catalyzed by a photoinitiator. This resulting hybrid material boasts a low viscosity and over 500 times the rigidity of the photoresist film. Moreover, it enables the attainment of pattern resolutions in the range of 100 nm to several microns when exposed to UV light—a capability well-suited for supporting the etching process in UV-NIL [82].

**Figure 12:**
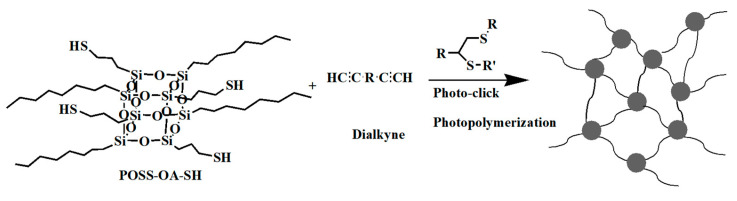
Schematic diagram of crosslinked compounds prepared by light click reaction between four-substituted SH-POSS and alkynes.

Drawing inspiration from thiol-alkene click chemistry principles, Lin H. et al. [83] introduced a mixed photoresist of thiol-alkynes in 2014, offering another avenue for designing UV-NIL photoresists. Illustrated in Figure 12, this approach involved the use of four-substituted mercaptopropyl POSS to engage in a light-click reaction with alkynes, yielding hybrid materials with a degree of cross-linking. These compounds exhibit excellent coating properties, thermal stability, resistance to oxygen plasma etching, low surface energy, and minimal volume shrinkage. Differing from previous SH-POSS-based photoresists, thiol-alkyne compounds possess higher relative molecular weights and lower volatility, thereby eliminating the characteristic odor of thiol monomers. The resulting hybrid materials resemble the thiol-ene system and excel at high-resolution pattern transfer onto silicon substrates. When polystyrene (PS) is employed as the transfer membrane, the original polymer’s height is preserved.

Incorporating diazenone and hydroxyl groups into POSS-based photoresists imparts several desirable characteristics, including high modulus, low shrinkage, remarkable transparency (95.8%), low surface energy, and resistance to organic solvents. When employed in NIL molds, diazenone-POSS photoresists offer distinct advantages over PDMS. PDMS often faces challenges in achieving nanometer-scale patterning due to its insufficient Young’s modulus. In contrast, diazenone-POSS demonstrates the capability to address this limitation and exhibits high tolerance to organic solvents during UV-NIL processes [84]. Compared to Young’s modulus, diazenone-POSS exhibits a significantly higher value of 3.626 GPa, surpassing PDMS by approximately 3.6 GPa [85]. Furthermore, the volume shrinkage of diazenone-POSS ranges from 4.3% to 6.9%. This behavior is attributed to the diazogroup’s release of small nitrogen gas molecules under UV irradiation. Following UV exposure, the resist’s overall swelling rate remains remarkably low, ranging between 0.11 and 0.53. This minimal swelling property effectively prevents unintended adhesion and ensures the reproducibility of UV-NIL processes.

**Figure 13:**
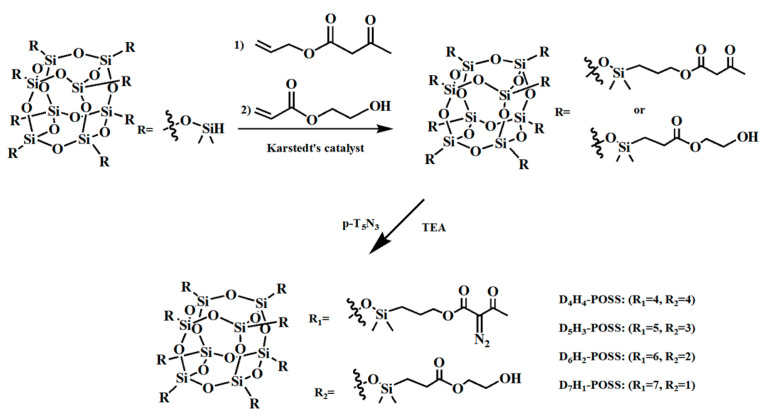
Preparation of DxHy-POSS corrosion materials with various chemical functions.

**Figure 14:**
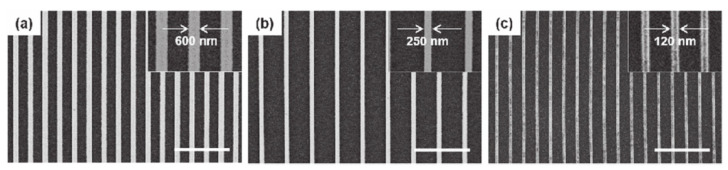
SEM images of the first replication mode of different dimensions with width, respectively (**a**) 600 nm, (**b**) 250 nm, and (**c**) 120 nm. Adapted from Ref. [86] with permission from IOP Publishing Ltd.

Diazenone-POSS-based photoresists form cross-linked resists, as depicted in Figure 13, allowing for faithful replication of patterns from silicon master plates. This replication occurs seamlessly across large areas without the introduction of defects. Consequently, this material simplifies the production of replica molds under mild conditions. Figure 14 presents SEM images showcasing the first replication of patterns of varying sizes. Notably, these images reveal uniform linear patterns with widths of 600 nm, 250 nm, and 120 nm, respectively, without any critical defects. Furthermore, when diazenone-POSS molds are employed in UV-NIL processes, they exhibit exceptional performance, devoid of attachment issues, pattern deformation, or variations in pattern height. Importantly, diazenone-POSS resists maintain pattern integrity throughout UV-NIL processing without any discernible shrinkage or expansion issues [86].

**Figure 15:**
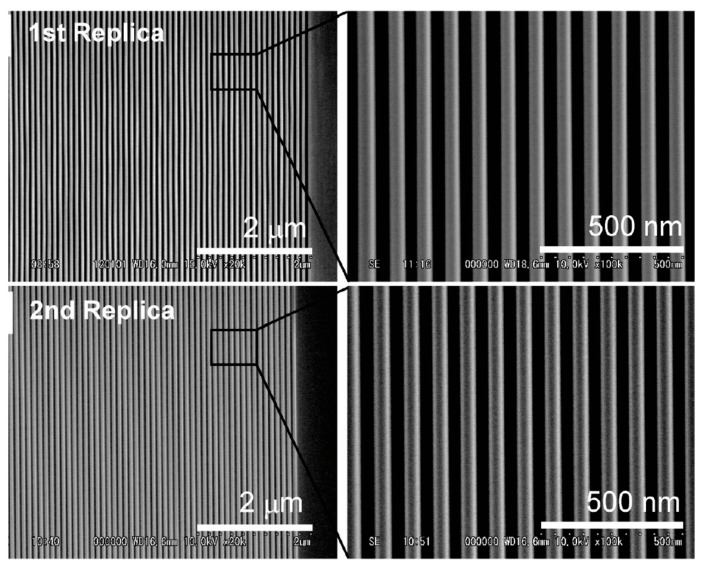
POSS graft mold prepared with fluoromethylacrylate compound for lithography verification: 1st Replica and 2nd Replica are the motifs of the UV-NIL experienced 20 times on the PET template and on the glass substrate, respectively. Adapted from Ref. [46] with permission from the American Chemical Society.

In addition to SH-POSS-based photoresists, there are alternative POSS-based photoresists that fulfill the requirements for NIL etching. For example, PMMA-POSS is suitable for NIL applications [46]. The viscosity-reducing PMMA-POSS formulation has the advantages of high modulus and minimal volume shrinkage and thus can be effectively used as a replica mold for photo printing testing. As depicted in Figure 15, the molds successfully underwent 20 UV-NIL cycles, effectively reproducing sub-50 nm characteristic lines and spatial patterns on both PET film and glass substrates without sustaining any damage.

**Figure 16:**
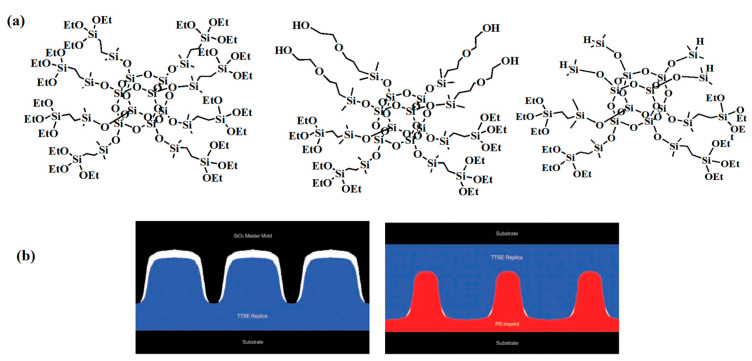
(**a**) The molecular structure diagram of POSS with different functional groups in the hard photoresist mold; (**b**) Parent plate: not washed with oxide buffer solution (**left**); the parent plate is washed with oxide buffer solution (**right**). Adapted from Ref. [87] with permission from WILEY-VCH Verlag GmbH & Co. KGaA.

As illustrated in Figure 16a, three distinct POSS molecules containing various functional groups were condensed through hydrolysis under acidic conditions to yield polymers characterized by a cross-linked network structure. These polymers possess dual utility, serving both as raw materials for NIL photoresists and as templates for embossed molds. Notably, they exhibit minimal shrinkage, exceptional thermal stability, impressive UV transmittance, and inherent low surface energy. Moreover, the wet etching of the master plate using an oxide buffer solution resulted in the reduction of line widths from 30 nm to 20 nm, as depicted in Figure 16b. Collectively, these examples underscore the significant potential of POSS-based materials as molds in NIL lithography [87].

UV-NIL offers a straightforward and cost-effective approach while exerting lower pressure compared to traditional NIL. Consequently, it facilitates the attainment of enhanced resolution within the photoresist layer. Despite economic challenges, UV-NIL remains competitive within the realm of lithography, primarily due to the unique characteristics of liquid POSS-based photoresist resins that undergo UV curing. Notably, UV-NIL has encountered certain issues, such as the occurrence of a “residual layer” and pattern collapse stemming from mold removal. These challenges have been successfully mitigated through the introduction of POSS, which enables precise regulation of the surface chemistry of composites and facilitates the selection of soluble molds. Additionally, High-Resolution Electron-Beam Sensitive Glass (HSQ) has been subjected to rigorous evaluation within the context of UV-NIL.

### 2.4. The DUV-POSS-Based Photoresist

Deep Ultraviolet (DUV) lithography is a technique employing wavelengths of 248 nm and 193 nm for precision patterning. A distinguishing characteristic of DUV lithography is its capacity for rapid focusing on surfaces of diverse geometries.

In an endeavor to assess the applicability of DUV lithography at varying fluorination levels, researchers grafted fluoromethylacrylate onto POSS to form a polymer [53]. Their investigations demonstrated that PMMA-POSS compounds, when used as resist agents, can achieve a longitudinal aspect ratio (L/S) of 1:1 or 1:2. When used as a transfer layer in bilayer photolithography, fluorinated PMMA-POSS facilitates the transfer of patterns to the substrate without causing pattern collapse or contamination. However, it is noted that microphase separation occurs within the film. This phenomenon may be attributed to the uneven resist behavior induced by the presence of acid produced by the varying lengths of the fluorinated chains, which alters the overall hydrophobicity of the photoresist system, thereby negatively affecting processes like baking and development. Furthermore, the presence of PAG monomers in the photoresist results in the formation of insoluble surface layers and colloidal contamination, contributing to line width deterioration and limiting resolution [88,89,90].

**Figure 17:**
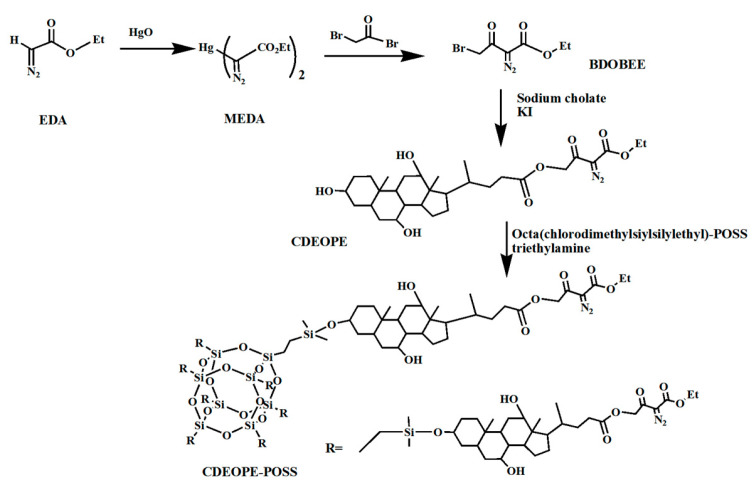
Chemical structure of the CDEOPE-POSS.

**Figure 18:**
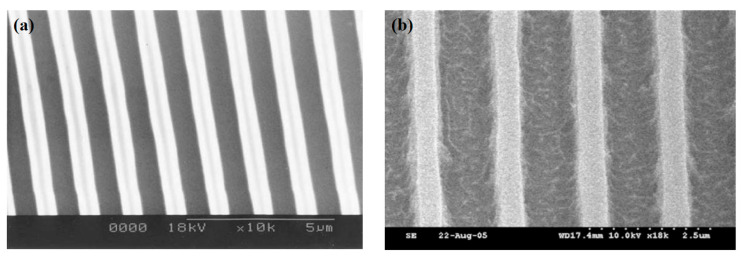
(**a**) Octa(chlorodimethylsilylethyl)-POSS hydrogenosanized with diazone one, single-layer DUV lithography pattern of resist (**b**) Octa(chlorodimethylsilylethyl)-POSS and diazone one, double-layer DUV lithography pattern of resist. Adapted from Ref. [91] with permission from The Royal Society of Chemistry.

In contrast, the diazonium group does not generate PAG monomers in the DUV exposure zone, and POSS, acting as a high-silicon content component, covalently bonds with the diazonium group, thereby conferring favorable lithographic performance. Diazenone-POSS also offers greater ease of functionalization. In 2006, Jin-Baek Kim et al. [91] synthesized octa(chlorodimethylsilylethyl)-POSS (CDEOPE-POSS, Figure 17) modified with bile acid(diazonium) ester. Subsequently, they subjected the resist to monolayer and bilayer DUV lithography, causing the development of 2.38 wt% TMAH. 0.7 μm lines were obtained at doses of 300 mJ·cm^−2^ and 250 mJ·cm^−2^, respectively, as depicted in Figure 18.

Currently, DUV lithography remains a prominent technology within the lithography domain, albeit with notable concerns arising from the exposure process involving potentially hazardous chemical agents [91,92]. These concerns encompass the toxicity of organic solvents, the adverse effects of alkaline developers, and concerns regarding carcinogenicity, both to individuals conducting experiments and to the broader environment [93,94,95].

As a silicon-based hybrid material, POSS necessitates enhancements to address its performance limitations in the realm of DUV lithography. In addition to addressing the organic solubility of POSS, attention must also be directed toward challenges related to substrate adhesion and the distribution of relative molecular weights within POSS polymers [96,97]. These endeavors are crucial for advancing the compatibility and effectiveness of POSS-based photoresists in the DUV lithography domain.

The most commonly used field of DUV lithography is CAR, which often requires PAG to trigger the generation of photoresists, and the formula of photoresists is more complex. Due to the presence of PAG, lithographic patterns may suffer from acid diffusion, which can degrade the quality of the lithographic pattern during development. POSS-based resin can be molded and formulated (without post-baking) as a photoresist resin without PAG, which also has a wide processing temperature range. Therefore, the combination of the development in photoresist materials and the wavelength of the lithography light source can promote the progress of semiconductor nano-processing technology in an essential direction.

### 2.5. The EUV-POSS-Based Phtotresist

EUV lithography is a cutting-edge technique employed for etching intricate patterns onto the surface of a photoresist film using extreme ultraviolet light with a wavelength of 13.5 nm. EUV lithography sets demanding performance criteria, necessitating a sensitivity level below 10 mJ·cm^−2^, resolution finer than sub-10 nm, and line edge roughness of less than 1.5 nm. Nevertheless, achieving these stringent requirements becomes increasingly challenging, especially when dealing with ultra-high energy EUV lithography, particularly when working with ultra-thin or surface imaging photoresists, which can compromise resolution [98].

In response to these challenges, a series of innovative approaches have been explored. In 2003, M. Azam Ali and colleagues introduced a novel chemically amplified resist (CAR), demonstrating exceptional sensitivity to EUV light (Figure 6), with an impressive EUV sensitivity of up to 1.0 g·cm^−3^ for a film thickness of 100 nm [52]. Subsequently, in 2016, Daniel Fan and co-researchers achieved significant progress in EUV lithography by utilizing an Ir diffraction grating in place of the conventional Gaussian grating. This breakthrough resulted in the successful creation of 6 nm L/S patterns using EUV lithography [99].

In the realm of EUV lithography, the selection of silicon-based materials as photoresists is a strategic choice, primarily due to the alignment of the electron binding energy of the silicon core with the energy levels of EUV radiation [100].

**Figure 19:**
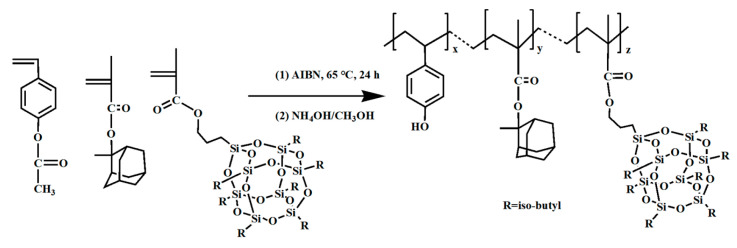
The synthetic route of p-hydroxystyrene-co-2-methyl-2-amantantl methacrylate-co-isoButyl methacrylate-POSS.

An innovative approach involved the copolymerization of acetyloxystyrene (AcOSty), methacrylic acid 2-methyl-2-ammanyl ester (MAdMA), methyl propylene isobutyl-POSS (MaIBPOSS), and 2,2′-azodiisobutyric nitrile (AIBN) as the initiator, yielding a new polymer incorporating POSS, as illustrated in Figure 19 [101]. This polymer underwent rigorous evaluation using EUV lithography and was developed with a TMAH developer, showcasing an exceptional sensitivity of 5 mJ·cm^−2^. In addition, scanning electron micrographs showed that 100 nm resist patterns could be successfully created at a dosage of 12 mJ·cm^−2^. The introduction of POSS into the polymer matrix effectively enhanced dry etching resistance and lithographic performance.

**Figure 20:**
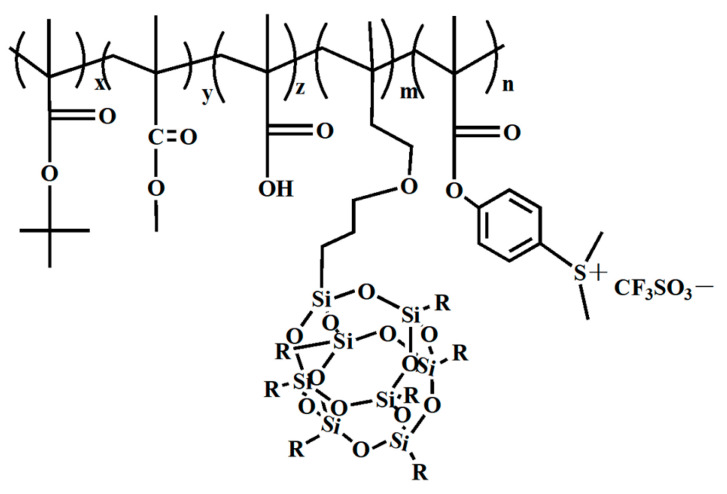
Nanocomposite photoresist (x = 9.0, y = 7.0, z = 1.0, m = 2.0, n = 1.0).

The synthesis of a nanocomposite photoresist combining POSS (*tert*-butyl methacrylate) t-BMA, MA, MMA, and PAG is illustrated in Figure 20. This photoresist exhibited excellent film-forming properties and proved highly efficient in EUV lithography when initiated. Exposure doses ranging from 1 to 1.5 mJ·cm^−2^ yielded patterns characterized by high contrast (3.0) and exceptional resolution, devoid of noticeable defects [52]. Additionally, HSQ was found to produce sufficient crosslinks at EUV doses within the range of 4000 to 8000 mJ·cm^−2^, achieving resolutions as fine as 6 nm [102]. Recent investigations into EUV lithography underscored that a characteristic size of 10 nm exhibited a sensitivity level of 1/2 at exposure doses ranging from 60 to 70 mJ·cm^−2^ [103].

In the realm of EUV lithography, despite its considerable prominence, the precise etching mechanism governing the interaction between the photoresist resin and EUV light remains an unresolved enigma. Research conducted by Rathore A. involving an EUV lithography machine utilizing HSQ unveiled significant insights. Notably, it was observed that T-type H-POSS caused the rupture of Si-O-Si and Si-H bonds within the POSS framework. Despite the application of a 2.38% *v*/*v* TMAH developer, the resulting polymer was unable to resolve the etching challenges associated with densely packed features. In contrast, the use of a more robust 5% *v*/*v* TMAH developer exhibited the capability to differentiate between compact features measuring 16 nm and 20 nm, albeit with accompanying Line Edge Roughness (LER) values exceeding 6 nm and 4.9 nm, respectively [58].

With the development of smaller photolithographic sizes, the alkaline developer is more likely to dissolve the pattern in the exposure area, and the development of water-soluble photoresist can further improve the effect of the alkaline developer on the photolithographic pattern [104]. Water-soluble photoresists have been investigated to reduce the use of toxic organic solvents and the generation of photolithography process waste, e.g., chemically modified chitosan and new styrene polymers [105,106]. In addition, EUV resists the need to have a higher absorption intensity for extreme ultraviolet light [107]. Si is an element with a strong absorption of EUV; POSS is just a silicon-rich compound. Water-soluble polymers of POSS composition can be synthesized by controlling the concentrations of octa-aminopropyl POSS (A-POSS) and octa-substituted carboxy-terminal POSS (C-POSS) [94]. Although there is currently no literature on water-soluble POSS-based photoresists, we believe that water-soluble POSS-based photoresists with highly extreme UV absorption could be a good solution. Therefore, research into water-soluble POSS photoresists is expected to mitigate the harmful effects of toxic reagents on human health and reduce the burden on the environment.

Overall, EUV lithography has garnered substantial attention in lithography technology due to its potential for achieving exceptional resolution. However, certain limitations inherent to EUV photoresist resins have impeded progress, particularly in the context of resolution and other critical parameters. Currently, EUV lithography has reached a 7 nm process node, but as feature sizes continue to diminish, the selection of suitable photoresist materials becomes the foremost impediment [7]. In this context, POSS-based silicone resin, owing to its inherent properties and the unique characteristics it imparts when combined with other compounds, emerges as a promising candidate for further exploration in the domain of EUV lithography. Its potential to address the specific challenges associated with EUV lithography positions it as a valuable avenue for future research and development efforts.

### 2.6. Directed Self-Assembly (DSA)-POSS-Based Photoresist

Directed Self-Assembly (DSA) represents a novel technique harnessing external fields to orchestrate the self-organization of intricate patterns. DSA lithography materials encompass an array of constituents, including block copolymers, molecular brushes, and liners. For instance, Li and Huck have successfully synthesized block copolymers (BCPs), specifically polystyrene-block-polymethyl methacrylate (PS-b-PMMA), which can serve as the very agents directly employed in NIL processes. Moreover, it is important to acknowledge that the peripheral organic groups of POSS cores exert a profound influence on the molecular behavior of POSS in hybrid materials [108]. This characteristic implies that polymer copolymers embedded with POSS can function as suitable candidates for DSA assembly, effectively synergizing DSA and lithography technologies. Such collaboration holds promise for advancing resolution and pattern density within photoresist materials, presenting a compelling alternative avenue for both DSA and photoresist research.

**Figure 21:**
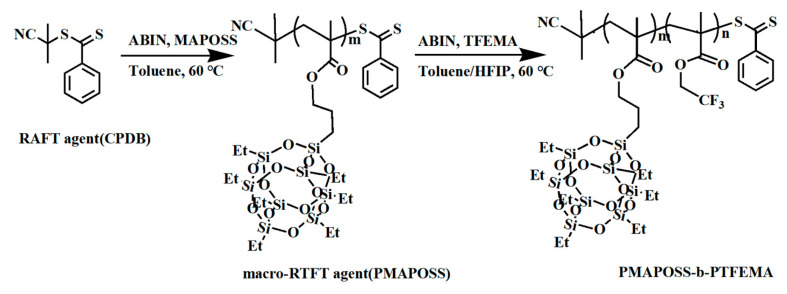
Synthetic scheme of PMAPOSS-b-PTFEMA.

**Figure 22:**
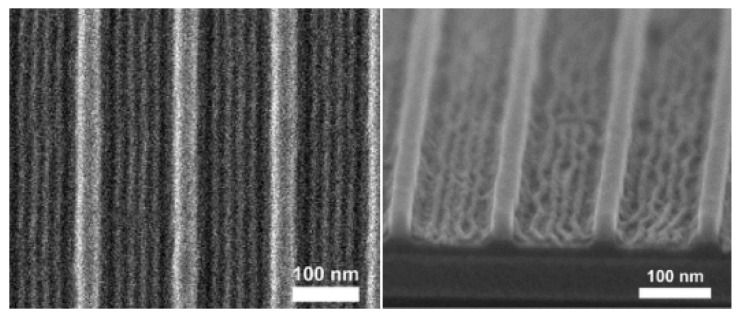
PMAPOSS-b-PTFEMA through ArF lithography TEM (**Left**, before O_2_/RIE); SEM (**Right**, after O_2_/RIE). Adapted from Ref. [109] with permission from the American Chemical Society.

**Figure 23:**
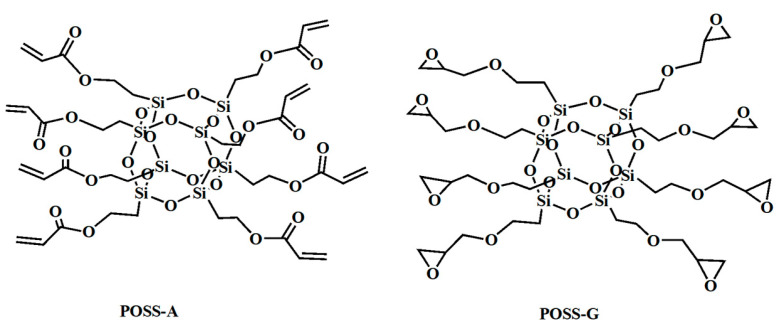
The chemical structure of POSS-A and POSS-G.

Nakatani R. et al. [109] leveraged poly(polyhedral oligomeric silsesquioxane methacrylate-block-2,2,2-trifluoroethyl methacrylate) (PMAPOSS-b-PTFEMA) (see Figure 21) in their research. Employing ArF lithography, they prepared a pre-patterned substrate featuring a half spacing of 8 nm. This demonstrated the compatibility of PMAPOSS-b-PTFEMA with existing DSA technologies. As is evident in Figure 22, TEM and SEM images of PMAPOSS-b-PTFEMA following ArF lithography depicted the capacity of Block Copolymers (BCPs) to form sub-10 nm sheet layers through straightforward annealing. Meanwhile, Borah D. [110] explored the utilization of Acrylate POSS (POSS-A) and Epoxy POSS (POSS-G) (see Figure 23) on a polystyrene-block-polydimethylsiloxane (PS-b-PDMS) substrate for guided self-assembly. This led to the creation of patterns corresponding to BCPs following UV-NIL. The incorporation of POSS-BCPs allowed for precise control over pattern orientation and alignment, culminating in the generation of silicon nanowires and cylindrical structures. The direction of orientation was determined by selectively removing or retaining the NIL residue layer [111]. This innovative fabrication method holds promise, particularly since it permits the formation of nanostruts and nanowire structures on the same substrate through the strategic removal of specific regions.

It is noteworthy that while the application of POSS-based photoresist resins in DSA lithography research remains relatively nascent, it offers a promising avenue for future exploration. Furthermore, it is worth mentioning that certain existing POSS compounds also exhibit potential for utilization in step-flash lithography and holographic lithography [112,113], hinting at additional directions for the investigation of POSS-based photoresist materials.

Finally, some of the factors mentioned in the text limiting the performance of the POSS-based photoresist are summarized in Table 2.

## 3. Conclusions

This review summarizes the evolutionary prospects of POSS and POSS matrix composites within the field of photoresist materials. We have analyzed several lithography systems, including electron-beam lithography (EBL), X-ray lithography (XRL) up to UV-NIL, deep ultraviolet (DUV) lithography, extreme ultraviolet (EUV) lithography, and DSA lithography. Through systematic studies, we discuss in detail the key properties of post-based photoresists, including membrane formation, sensitivity, resolution, solubility, and edge roughness.

HSQ (T-type H-POSS) exhibits high resolution and low edge roughness mode in EBL, and its universality throughout the lithography system lays the foundation for the application of POSS. In the field of X-ray lithography, POSS-based photoresists show promise in improving resolution and sensitivity, although there is still room for improvement. For example, the incorporation of fluorinated POSS into the acrylic resin effectively addresses the challenges of adhesion and phase separation. In UV nano-imprint lithography (UV-NIL) and thermal nano-imprint lithography (T-NIL), post-based photoresist is a compelling approach for continuous exploration and optimization. This diversification highlights the adaptability of POSS in different NIL environments.

The complexity of the high-resolution mode of DUV lithography and the role of the post-based photoresist in this context will also be discussed. The integration of POSS into the photoresist matrix not only improves the lithography performance but also leads to the observation of problems such as microphase separation within, necessitating further understanding of the interactions between different chemical components.

One of the limitations to the remarkable resolution of EUV light sources is the photoresist material, and POSS-based photoresists have the potential to improve sensitivity and resolution. Currently, water-soluble POSS-containing compounds may be a new way to provide a safer and more environmentally friendly method for EUV lithography.

DSA lithography represents an exciting frontier for post-embedded polymer copolymers with some compatibility with existing DSA technologies. As such, POSS has the ability to modulate material behavior and promote the assembly of complex patterns, thereby helping to improve the resolution and pattern density of photoresist materials.

With the continuous development of science and technology, miniaturization has become increasingly important. In existing studies, the application of photographic agents in step-flash lithography and holographic lithography offers exciting possibilities. Therefore, POSS-based lithography is worthy of further exploration by researchers and offers the potential to open up new areas of semiconductor manufacturing and nanotechnology.

## Figures and Tables

**Table 1:** POSS/MMA/TBA scale table (The arrows represent the correspondence between POSS and MAA/TBA).

POSS (wt.%)	MMA/TBA (wt.%/wt.%)				
	66.6/33.3	62/36	60.6/30.3	58.8/29.4	57.1/28.6
0	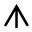 POSS0	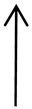	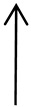	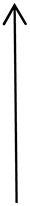	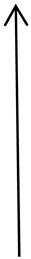
2		POSS1			
9.1			POSS2		
9.6	POSS8				
11.8	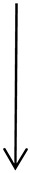	POSS5		POSS3	
14.3		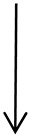	POSS6		POSS4
16.7			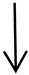	POSS7 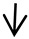	
POSS (wt.%)	89.5/0.9	88.2/0	85.7/0	83.3/0	
	MMA/TBA (wt.%/wt.%)				

**Table 2:** Summary of limitations of POSS-based photoresists in the text.

Some Limiting Factors for the POSS-Based Photoresist Resin			
Lithography System	POSS-Based Photoresists	Limitations	References
EBL	HSQ (T-type-POSS)	the chemical stability; the process of storage; the green issue of sustainability	[13,48,58]
X-ray	PMMA-POSS; POSS/MMA	thermal effects and signal integrity	[47,59,61,67,68]
UV-NIL	HSQ; epoxy-functionalized POSS;SH-POSS; PMMA-POSS; diazenone-POSS	economic challenges; “residual layer” and pattern collapse stemming from mold removal	[46,77,79,81,87,88]
DUV	CDEOPE-POSS	PAG diffusion	[91]
EUV	MaIBPOSS; HSQ	certain limitations inherent to EUV photoresist resins	[7,101,102,103]
DSA	PMAPOSS-b-PTFEMA; POSS-A; POSS-G	the application of POSS-based photoresist resins in DSA lithography research remains relatively nascent	[109,110]

## Data Availability

Not applicable.

## Abbreviations

All molecular abbreviations and their full names	
HSQ (T-type H-POSS)	hydrogen silsesquioxane
POSS	polyhedral oligomeric silsesquioxane
PMMA	polymethyl methacrylate
TMAH	tetramethylammonium hydroxide
AQM-HSQ	applied quantum materials-HSQ
PAG	photosensitive acid-producing agents
HMDS	hexamethyldisilazane
APSQ	acetylated phenylsilsesquioxane oligomer
MA	methacrylic acid
Vi-POSS	octamer half-siloxane
MMA	methyl methacrylate
PMMA-POSS	polymethyl methacrylate-POSS
SH-POSS	sulfhydryl functionalization-POSS
TMPT	trihydroxymethylpropane triacrylate
PS	polystyrene
PDMS	polydimethylsiloxane
TBA	tert-butyl acrylate
CDEOPE-POSS	octa(chlorodimethylsilylethyl)-POSS
AcOSty	acetyloxystyrene
MAdMA	methacrylic acid 2-methyl-2-ammanyl ester
MaIBPOSS	methyl propylene isobutyl-POSS
AIBN	2,2′-azodiisobutyric nitrile
t-BMA	*tert*-butyl methacrylate
LER	line edge roughness
DSA	directed self-assembly
BCPs	block copolymers
PS-b-PMMA	polystyrene-block-polymethyl methacrylate
PMAPOSS-b-PTFEMA	poly(polyhedral oligomeric silsesquioxane methacrylate-block-2,2,2-trifluoroethyl methacrylate)
BCPs	block Copolymers
POSS-A	acrylate POSS
POSS-G	epoxy POSS
PS-b-PDMS	polystyrene-block-polydimethylsiloxane
POSS-BCPs	POSS-block copolymers
XRL	X-ray lithography
DUV-NIL	deep ultraviolet nanoimprint lithography
DSA	guided self-assembled lithography
EUV	extreme ultraviolet lithography
UV	ultraviolet
DUV	deep ultraviolet
EBL	electron Beam Lithography
NIL	nanoimprint lithography
UV-NIL	ultraviolet nanoimprint lithography
CAR	chemically amplified resist
T-NIL	thermal nano-imprint lithography
A-POSS	octa-aminopropyl POSS
C-POSS	octa-substituted carboxy-terminal POSS
SH-POSS	sulfhydryl functionalization-POSS
